# Mapping corpus callosum surface reduction in fetal alcohol spectrum disorders with sulci and connectivity-based parcellation

**DOI:** 10.3389/fnins.2023.1188367

**Published:** 2023-06-09

**Authors:** Justine Fraize, Gabrielle Convert, Yann Leprince, Florent Sylvestre-Marconville, Eliot Kerdreux, Guillaume Auzias, Julien Lefèvre, Richard Delorme, Monique Elmaleh-Bergès, Lucie Hertz-Pannier, David Germanaud

**Affiliations:** ^1^UNIACT, NeuroSpin, Frederic Joliot Institute, Centre d'études de Saclay, CEA Paris-Saclay, Gif-sur-Yvette, France; ^2^InDEV, NeuroDiderot, Inserm, Université Paris Cité, Paris, France; ^3^Institut de Neurosciences de La Timone, CNRS, Aix-Marseille Université, Marseille, France; ^4^Department of Child and Adolescent Psychiatry, Robert-Debré Hospital, AP-HP, Centre of Excellence InovAND, Paris, France; ^5^Department of Pediatric Radiologic, Robert-Debré Hospital, AP-HP, Centre of Excellence InovAND, Paris, France; ^6^Department of Genetics, Robert-Debré Hospital, AP-HP, Centre de Référence Déficiences Intellectuelles de Causes Rares, Centre of Excellence InovAND, Paris, France

**Keywords:** fetal alcohol spectrum disorder (FASD), corpus callosum, segmentation, cortical sulci, connectivity, microcephaly

## Abstract

**Introduction:**

Fetal alcohol spectrum disorders (FASD) range from fetal alcohol syndrome (FAS) to non-syndromic non-specific forms (NS-FASD) that are still underdiagnosed and could benefit from new neuroanatomical markers. The main neuroanatomical manifestation of prenatal alcohol exposure on developmental toxicity is the reduction in brain size, but repeated imaging observations have long driven the attention on the corpus callosum (CC), without being all convergent. Our study proposed a new segmentation of the CC that relies on both a sulci-based cortical segmentation and the “hemispherotopic” organization of the transcallosal fibers.

**Methods:**

We collected a monocentric series of 37 subjects with FAS, 28 with NS-FASD, and 38 with typical development (6 to 25 years old) using brain MRI (1.5T). Associating T1- and diffusion-weighted imaging, we projected a sulci-based cortical segmentation of the hemispheres on the midsagittal section of the CC, resulting in seven homologous anterior–posterior parcels (frontopolar, anterior and posterior prefrontal, precentral, postcentral, parietal, and occipital). We measured the effect of FASD on the area of callosal and cortical parcels by considering age, sex, and brain size as linear covariates. The surface proportion of the corresponding cortical parcel was introduced as an additional covariate. We performed a normative analysis to identify subjects with an abnormally small parcel.

**Results:**

All callosal and cortical parcels were smaller in the FASD group compared with controls. When accounting for age, sex, and brain size, only the postcentral (η^2^ = 6.5%, p_FDR_ = 0.032) callosal parcel and % of the cortical parcel (η^2^ = 8.9%, p_FDR_ = 0.007) were still smaller. Adding the surface proportion (%) of the corresponding cortical parcel to the model, only the occipital parcel was persistently reduced in the FASD group (η^2^ = 5.7%, p_FDR_ = 0.014). In the normative analysis, we found an excess of subjects with FASD with abnormally small precentral and postcentral (peri-isthmic) and posterior–splenial parcels (p_FDR_ < 0.05).

**Conclusion:**

The objective sulcal and connectivity-based method of CC parcellation proved to be useful not only in confirming posterior–splenial damage in FASD but also in the narrowing of the peri-isthmic region strongly associated with a specific size reduction in the corresponding postcentral cortical region (postcentral gyrus). The normative analysis showed that this type of callosal segmentation could provide a clinically relevant neuroanatomical endophenotype, even in NS-FASD.

## 1. Introduction

The pathological consequences of prenatal alcohol exposure (PAE) are grouped under the diagnosis of fetal alcohol spectrum disorders (FASD) that range from fetal alcohol syndrome (FAS) to non-syndromic, non-specific forms (NS-FASD) (Astley, 2004; Cook et al., 2016; Hoyme et al., 2016). The positive diagnosis of FAS is based on a consensual set of clinical features, including facial dysmorphia, growth retardation, and microcephaly, while the positive diagnosis of NS-FASD remains probabilistic. One of the main targets of the teratogenic effects of ethanol is the brain; subjects with FASD tend to have not only a smaller brain but also recurrent focal brain abnormalities detectable with magnetic resonance imaging (MRI). However, no specific neuroanatomical criterion has been added to the diagnostic guidelines.

One of the more frequently reported of these focal brain abnormalities occurs in the corpus callosum (CC) (for reviews, see: Lebel et al., 2011; Donald et al., 2015; Nguyen et al., 2017; Moore and Xia, 2021). In Astley et al.'s cohort of 65 children with FASD (6 to 16 years old), two had hypoplasia or agenesis of the CC (Astley et al., 2009). Autti-Rämö et al. described two adolescents of approximately 14 years among the 17 adolescents who were prenatally exposed to alcohol with anomalies of the CC (one thinning and one hypoplasia) (Autti-Rämö et al., 2002). Similarly, Boronat et al. (2017) listed among the 62 subjects aged between 4 and 18 years old with FASD, 24 with hypoplasia of the CC, and two with partial agenesis. Recently, Treit et al. (2020) described five subjects with dysmorphic CC, associated with the asymmetry of the lateral ventricles among 124 subjects with FASD from school age to adulthood. In a previous radiological-clinical study, we reported two cases of partial agenesis of the CC over 89 subjects with FASD from school age to early adulthood and described, with manual measurements and normative scaling analysis, an isthmus narrowing as a recurrent abnormality in both FAS and non-syndromic FASD (Fraize et al., 2022). In addition to these radiological descriptions, computational neuroimaging has searched for more subtle anomalies or more objective morphometric descriptions. Several studies of the CC in the FASD population recommended a reduction in the midsagittal CC area (Riley et al., 1995; Sowell et al., 2001; Astley et al., 2009; Dodge et al., 2009), thickness (Yang et al., 2012), volume (Gautam et al., 2014; Biffen et al., 2020; Inkelis et al., 2020), or shape with a flattened or misshapen appearance (Sowell et al., 2001; Bookstein et al., 2002) that could be correlated to the amount of prenatal alcohol consumption (Biffen et al., 2017; Jacobson et al., 2017). Within the callosal structure itself, it appears that the size reduction affected the posterior region more severely, notably the isthmus and the splenium size (Sowell et al., 2001; Dodge et al., 2009; Fraize et al., 2022) or position (Sowell et al., 2001; Bookstein et al., 2002). Note that while several studies of the last 20 years, mostly in children and young adults, tend to converge toward a global reduction in the size of the CC, affecting its posterior part in particular, this result has proved difficult to fully or systematically replicate, even within large cohorts (Sowell et al., 2001; Yang et al., 2012; Marshall et al., 2022).

The CC is a large white matter fiber bundle consisting of axonal projections between homologous cortical regions and crossing the interhemispheric plane as a compact well-individualized midsagittal structure. Midsagittal CC fibers mainly have a “hemispherotopic” organization and two adjacent CC fibers corresponding to two adjacent points on the hemisphere (Pandya et al., 1971; Friedrich et al., 2020), and the frontal connectivity is largely overrepresented, accounting for the anterior two-third of the structure (Park et al., 2008; Chao et al., 2009; Wang et al., 2020). At first approximation, the midsagittal section of the CC is often proposed as a proxy for the much larger and more complex 3D whole CC bundle. It can be divided into several regions to be compared across subjects in terms of area, length, or thickness. These divisions are commonly determined by neuroradiologists based on the arbitrary geometrical processes deemed to guarantee homology between subjects (Witelson, 1989; Hofer and Frahm, 2006). Relying only on the midsagittal anatomy or geometry, such approaches are not related to the actual anatomical connectivity. In fact, they may be not only irrelevant in the case of the pathological shortening (partial agenesis of the CC) that occurs in FASD but also insensitive to the interindividual variations of relative cortical representation in the midsagittal section. Even if the results are still ambiguous concerning FASD-associated regional anomalies of hemispheric volume (Archibald et al., 2001; Astley et al., 2009), cortical thickness (Sowell et al., 2008; Zhou et al., 2011; Yang et al., 2012; Treit et al., 2014; Marshall et al., 2022), or extension (Rajaprakash et al., 2014; Hendrickson et al., 2018), any midsagittal anomaly of the CC should raise questions about either cortical or white matter damage (Gautam et al., 2014; Fan et al., 2016; Ghazi Sherbaf et al., 2019; Kar et al., 2022), prompting a conjoint analysis of both cortical and callosal anatomy. Hence, it appears important to introduce and use parcellation of the CC into as many parcels as relevant in the regions defined on the cortical surface anatomy and projected on the midsagittal section through the real hemispherotopic callosal–cortical connectivity. A few implementations have been proposed during the past 20 years (Huang et al., 2005; Styner et al., 2005; Chao et al., 2009) without resulting in a consensual use for callosal parcellation and morphometry, but they have never been applied to the study of developmental conditions, let alone FASD.

Therefore, we aimed to describe the anatomy of the midsagittal section of the CC in a large monocentric cohort of FASD and typically developing control subjects, by means of its automatic anterior–posterior parcellation into seven parcels, achieved at the individual level from T1- and diffusion-weighted images, reflecting a fan-shaped segmentation of the cortex along six major sulcal meridians. The comparison between FASD or FAS and typical callosal anatomy was performed not only by considering covariates, including the cortical extension, as a brain size proxy but also a relative representation of the reference cortical parcel to better disentangle pathological phenomena and increase sensitivity in the detection of FASD singularity. In addition to the group comparison, we completed the analysis of our models of the callosal surfaces by a normative approach to show the detectability at the individual level of likely focal callosal anomalies.

## 2. Material and methods

### 2.1. Population and MRI data

A total of 65 subjects with FASD, aged between 6 and 25 years, were included retrospectively from a clinical series of patients with neurodevelopmental disorders who were admitted to the Child Neurology Department at Robert-Debré University Hospital between 2014 and 2020. FASD diagnosis was established using the two main guidelines (Astley, 2004; Hoyme et al., 2016), and a full differential diagnosis work-up was completed, notably a systematic brain MRI. The exclusion criteria were participants who were prenatally exposed to another embryo-fetotoxic agent or who explicitly disagreed with the study participation. Finally, subjects with FASD were separated into two groups: the syndromic or FAS (including partial FAS) and the non-syndromic or NS-FASD. The clinical and radiological characteristics of the 65 subjects with FASD are detailed in Table 1. This cohort and the diagnostic procedure were already described in a previous study (Fraize et al., 2022, 2023).

**Table 1:** Sociodemographic, clinical, radiological data of subjects with FASD.

	**FAS**	**NS-FASD**	***p*-value**
n =	**37**	**28**	
**Sociodemographic assessment**			
Sex: male *n* (%)	17 (47.2)	15 (57.7)	0.578
Age at MRI, mean in years (SD)	11.17 (3.72)	13.06 (4.97)	0.098
**Clinical assessment**, ***n*** **(%)**			
**(1) Prenatal alcohol exposure**			
4.Confirmed, severe	15 (40.5)	13 (46.4)	0.825
3.Confirmed, moderate or unquantified	20 (54.1)	13 (46.4)	0.720
2.Not documented	2 (5.4)	2 (7.1)	1.000
1.No exposition	0 (0.0)	0 (0.0)	-
**(2) FAS facial features**			
4.Severe	22 (59.5)	2 (7.1)	**< 0.001**
3.Moderate	15 (40.5)	0 (0.0)	**< 0.001**
2.Mild	0 (0.0)	23 (82.1)	**< 0.001**
1.None	0 (0.0)	3 (10.7)	0.075
**(3) Growth deficiency**			
4.Significant	14 (37.8)	3 (10.7)	**0.020**
3.Moderate	8 (21.6)	2 (7.1)	0.175
2.Mild	6 (16.2)	6 (21.4)	1.000
1.None	9 (24.3)	17 (60.7)	**0.012**
**Brain anatomy**			
(4) Structural central nervous system damage	29 (78.4)	17 (60.7)	0.097
Head circumference, smallest known			
(4) ≤ - 2 SD: microcephaly	26 (70.3)	14 (50.0)	0.086

Diagnostic criteria of the 4-Digit Diagnostic Code are numbered from (1) to (4). FAS, fetal alcohol syndrome; FASD, fetal alcohol spectrum disorder; NS-FASD, non-syndromic FASD; SD, standard deviation. In bold, *p*-values < 5%.

A total of 38 typically developing subjects, aged between 6 and 25 years, with no report of PAE, developmental delay, or family history of the neurological or psychiatric condition (1^st^ degree) were included for comparison (control group), as part of a research program on autism in the Department of Child and Adolescent Psychiatry of Robert-Debré University Hospital. There were no significant sex or age differences in the control groups compared with the FASD group (*p* = 0.813 and *p* = 0.479 respectively) (Table 2).

**Table 2:** Clinical characteristics of the two groups of subjects.

	**Controls**	**FASD**	***p*-value**
***n***	**38**	**65**	
Sex: male *n* (%)	17 (0.45)	32 (0.49)	0.813^†^
Age at MRI, mean in years (SD)	12.63 (4.15)	12.02 (4.37)	0.479^††^

FASD, fetal alcohol spectrum disorder; SD, standard deviation.^†^*p*-value of the comparison of proportions for sex,^††^*p*-value of the comparison of means by Student's *t*-test.

MRI data acquisition was performed in the Department of Pediatric Radiology of Robert-Debré University Hospital on the same 1.5T scanner (Ingenia, Philips Healthcare, Amsterdam, the Netherlands) including a 3D T1-weighted FFE-TFE sequence (1 mm isotropic; TR = 8.2ms; TE = 3.8ms; TI = 0.8s; Flip = 8°; SENSE = 2), a 2D diffusion-weighted SE-EPI (2.5 mm isotropic, b1,000 s/mm2, 32 directions), and a field map. A visual quality check was systematically performed to exclude images of insufficient quality.

### 2.2. Image processing

We combined the existing tools for sulcal-based cortical segmentation and tractography to perform an original parcellation of the midsagittal section of the CC into seven parcels along the anterior–posterior axis, achieved at the individual level in the native space without any template-based normalization process. The delineation and further inter-individual homology of the cortical and callosal parcels rely on the identification of major sulcal landmarks previously described as “sulcal meridians” (Auzias et al., 2013).

**Figure 1:**
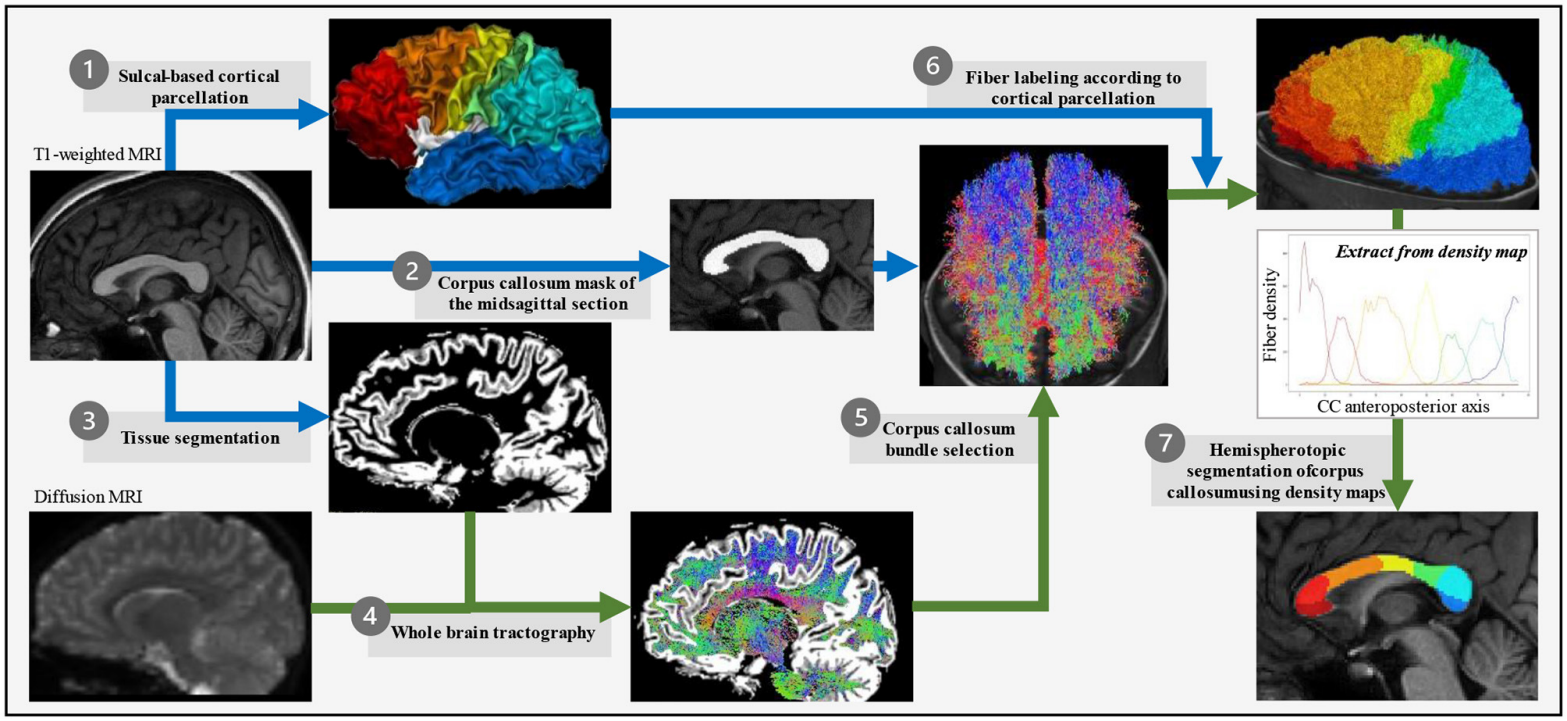
Corpus callosum parcellation pipeline. For each subject in its own image space, T1-weighted images were used to obtain the cortical surface (*Morphologist*) that was then divided into seven parcels based on the *Hip-Hop* model of geodesic representation of the cortex (adapted from *MarsAtlas*) **(1)**, the mask of the midsagittal section of the corpus callosum (*Morphologist*) **(2)**, and the white *vs*. gray matter segmentation (*FSL*) **(3)**. Diffusion-weighted images were used to generate the whole-brain tractography (*MRtrix3*) **(4)** from which the corpus callosum fiber bundle was selected for passing through the midsagittal section of the corpus callosum (*MRtrix3*) **(5)**, and then its fibers connecting two homologous parcels of the sulcal-based cortical parcellation labeled according to this bilateral homologous interhemispheric connectivity (*MRtrix3*) **(6)**. Finally, a regularized majority vote on the labeled-fibers density map allowed to assign a cortical-based label to each voxel of the midsagittal section of the corpus callosum **(7)**.

T1-weighted MRI data were processed within the *BrainVISA/Anatomist* (RRID:SCR_007354), using *Morphologist2015* (Rivière et al., 2009; Perrot et al., 2011) for tissue segmentation, inner cortical surface meshing, sulci modeling, and identification. All the MRI processing steps explained below are summed up in Figure 1.

#### 2.2.1. Midsagittal section of the corpus callosum

The mask of the midsagittal section of the CC was obtained within the *Morphologist2015* framework after oversampling of the T1-weighted images at 0.5 mm isotropic resolution, by intersecting the white matter mask with the midsagittal plane in the Talairach space, localizing the section in the interhemispheric plane. The mask was checked systematically for any obvious segmentation error that could be manually corrected.

#### 2.2.2. Sulcal-based parcellation of the cortical surface

The cortical parcellation was obtained individually for each subject in its own image space, with an adaptation of the *MarsAtlas* parcellation (Auzias et al., 2016), which is based on the *Hip-Hop* model (Auzias et al., 2013) of the geodesic representation of the cortex, where the CC wraps around a cingular pole and a set of well-identified major sulci are used as “meridians,” linking the cingular pole to the insular pole, and others as orthogonal “parallels.” We added the anterior (*aka* horizontal) ramus of the lateral fissure and anterior perpendicular ramus of the cingular sulcus (*aka* anterior vertical paracingulate sulcus) (Amiez et al., 2019) to the original *Hip-Hop* model to increase the constraints on the first and second sulcal meridian, respectively. The *Morphologist2015* identification and projection on the inner cortical surface of all the meridian sulci were systematically quality-checked and corrected for obvious errors by an expert (GD). Seven cortical parcels separated by the sulcal meridians were obtained by fusion of the original *MarsAtlas* parcels along the parallels, schematically corresponding to the following classical lobar and sub-lobar hemispheric regions: frontopolar, anterior prefrontal, posterior prefrontal, precentral, postcentral, parietal, and occipital. The corresponding cortical parcel surface area (CxPS, cm^2^) was computed, as well as the total cortical surface area (TCxS, cm^2^). The cortical parcellation and the binary mask of the midsagittal section of the CC were realigned to the diffusion space using FSL FLIRT (Jenkinson et al., 2002).

#### 2.2.3. Diffusion-weighted image processing and whole-brain tractography

Preprocessing of the diffusion-weighted image data was performed using tools from *FSL* (RRID:SCR_002823) (Jenkinson et al., 2012) and *MRtrix*3 (RRID:SCR_006971) (Tournier et al., 2019). FSL *eddy* (Andersson and Sotiropoulos, 2016; Andersson et al., 2016) corrects for motion, eddy currents, and susceptibility distortions related to the magnetic field B_0_, based on a field map acquired along with the diffusion. Fiber orientation distributions were estimated using the constrained spherical deconvolution method (Tournier et al., 2007). With the orientation distribution functions (ODF) thus obtained, the iFOD2 algorithm was used to perform probabilistic tractography (Tournier et al., 2010). We imposed biological constraints to optimize the quality of the tractograms, reducing the number of biologically aberrant “fibers” based on the segmentation of five distinct tissues of the T1-weighted image (Anatomically Constrained Tractography) (Zhang et al., 2001; Smith et al., 2012). A dynamic seeding strategy was used to generate 10^7^ streamlines (or fibers) per subject, and the *SIFT2* filter was employed to weigh streamlines according to their density (Smith et al., 2015).

#### 2.2.4. Hemispherotopic parcellation of the midsagittal section of the CC

The entire CC bundle was automatically extracted from the whole-brain tractography using the crossing of the midsagittal section of the CC as the positive selection criterion. Any CC fiber connecting two homologous parcels from our seven sulcal-based cortical parcellations was labeled accordingly, resulting in the segmentation of the CC into seven sub-bundles corresponding to its bilateral homologous interhemispheric connectivity. A volumetric fiber density map was then computed and restricted to the midsagittal section of the CC for each of the seven CC sub-bundles. Finally, a subdivision of the midsagittal section of the CC was obtained by assigning to each of its voxels the label of the cortical parcel that sent the most fibers by majority voting on density maps. A weighting of the vote by the eight nearest neighbors (half weight for the concerned voxel, a twelfth for the neighbors) was implemented to regularize a few isolated voxels observed with simple majority voting. The final parcellation of all subjects was quality-checked visually: No aberrant segmentation was observed, and no manual correction was performed. The final metric was the callosal surface area for the total CC surface and the frontopolar, anterior prefrontal, posterior prefrontal, precentral, postcentral, parietal, and occipital callosal parcel surface (CcPS, mm^2^). The code used to perform the selection of the CC bundle and the parcellation of the midsagittal section is released on https://github.com/neurospin/CC-parcellation-from-tractography.

### 2.3. Statistical analysis

Statistics were performed using R Project for Statistical Computing (RRID:SCR_001905), with a 5% alpha risk with both uncorrected (*p*-value) and corrected (*q*-value) FDR for multiple comparisons (Benjamini and Hochberg, 1995).

Group differences in clinical characteristics and callosal and cortical surface areas (CcPS and CxPS) were evaluated using two-sample *t*-tests for continuous variables and a chi-squared test for categorical variables.

To examine the surface area reduction in the callosal parcels (CcPS) in FASD subjects by comparison to typically developing controls, we performed multiple regression, including age at MRI, sex, and the TCxS as a proxy of brain size, using the following model (1):


(1) $$\begin{array}{l} CcPS=DIAG^{*}b_{DIAG}+SEX*b_{SEX}+AGE*b_{AGE}\\ \;+TCxS*b_{TCxS}+b_{0} \end{array}$$

where *b* is the unstandardized regression coefficient for each predictor.

Given the potential effect of FASD on the size of each cortical parcel surface area (CxPS), expressed in model (2) and their mechanical correlation with the corresponding callosal parcel surface area (CcPS), an additional model (3) was also tested, which included the CxPS expressed as a percentage of the total surface (*%CxSP* = *CxPS*/*TCxS*) as a covariate. The *p*-values and *t*-values associated with the unstandardized regression coefficients were used to assess the effect of each covariate and the eta-squared (η^2^) for the effect size.


(2) $$\begin{array}{l} \%CxPS=DIAG*b_{DIAG}+SEX*b_{SEX}+AGE*b_{AGE}\\ \;+TCxS*b_{TCxS}+b_{0} \end{array}$$

(3) $$\begin{array}{l} CcPS=DIAG*b_{DIAG}+SEX*b_{SEX}+AGE*b_{AGE}+TCxS*b_{TCxS}\\ \;+\%CxPS*b_{\%CxPS}+b_{0} \end{array}$$

For the normative analysis at the individual level, the model of CcPS as a function of sex, age, and brain size was regressed in the control group (4). The values of the regression coefficients (*b*_*AGE*/*control*_,_*b*_*SEX*/*control*_, *bTCxS*/*control*_) thus obtained were applied to the two FASD populations to obtain “residues” or deviations from the control model.


(4) $$\begin{array}{l} CcPS\left[control\right]=SEX*b_{SEX/control}+AGE*b_{AGE/control}\\ \;+TCxS*b_{TCxS/control}+b_{0} \end{array}$$

Finally, the numbers of subjects with FASD below the 10^th^ percentile of the control distribution, a threshold deemed of clinical relevance for potential anomaly detection, were tagged and counted and compared to the number of controls (Fisher's exact test).

## 3. Results

### 3.1. Anatomical analysis of CC parcellation

**Figure 2:**
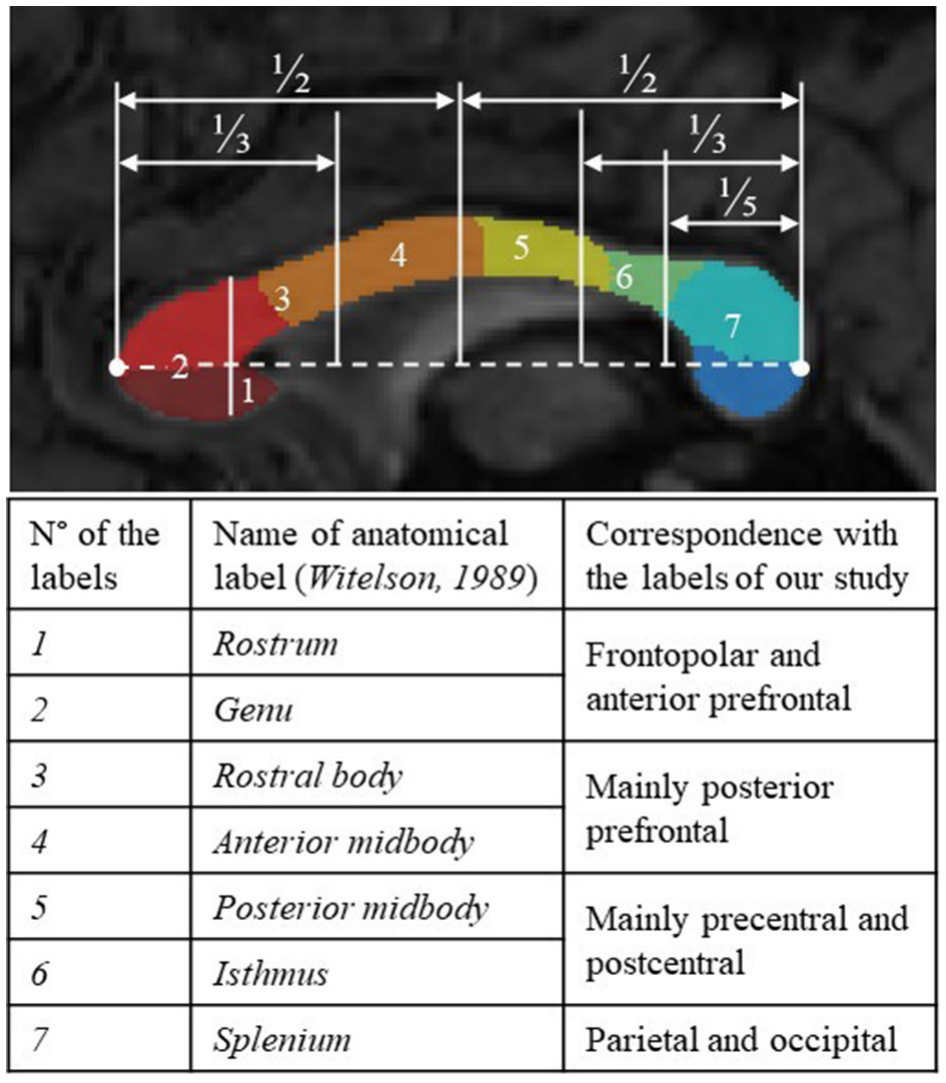
Qualitative comparison between arbitrary geometric parcellation of Witelson in 1989 with seven anatomical labels (delimitation appearing in white) and the parcels of our final segmentation (in color). Label numbers and names with a correspondence table.

**Figure 3:**
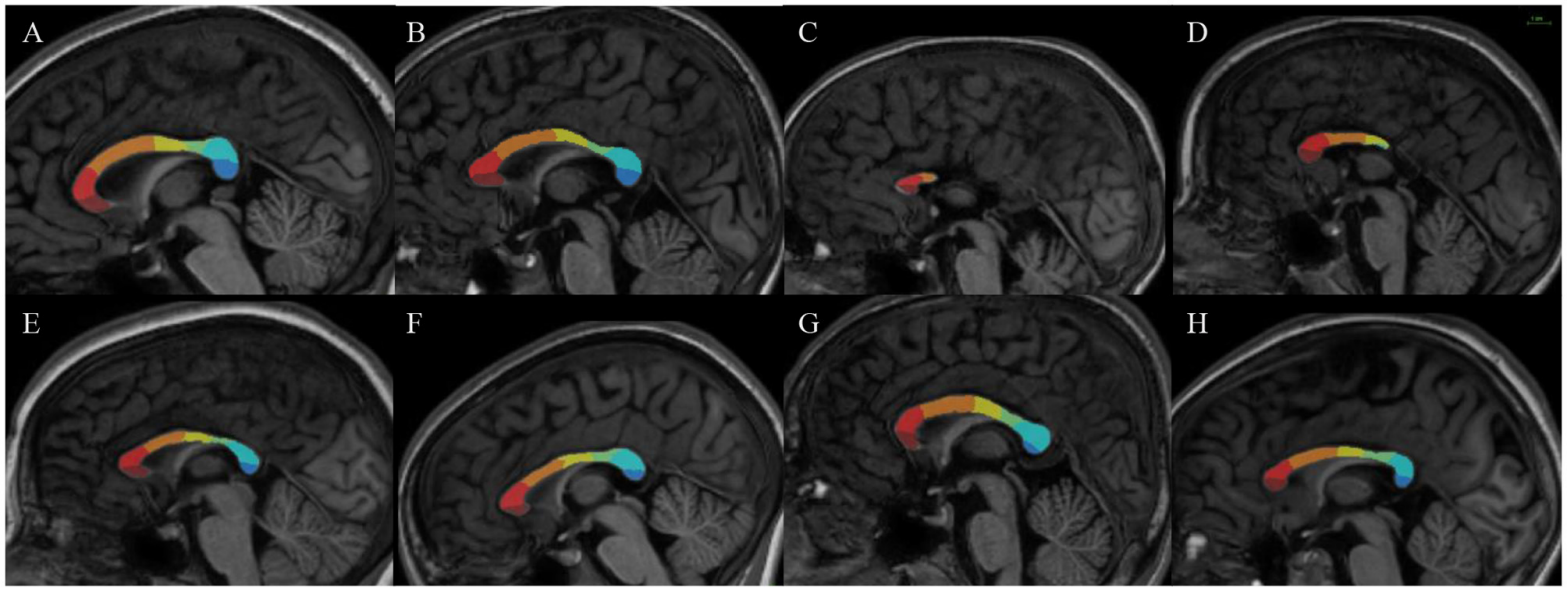
Results of our new segmentation pipeline of the corpus callosum in selected subjects, with the seven parcels (antero-posterior): frontopolar (dark red), anterior prefrontal (red), posterior prefrontal (orange), precentral (yellow) postcentral (green), parietal (light blue), occipital (dark blue). **(A, B)** Parcellation on control subjects. **(C, D)** Subjects with FAS with partial agenesis of the corpus callosum. **(E)** Subject with FAS. **(F–H)**. Subjects with NS-FASD. The scale common to all images appears at the top right.

For all the FASD and control subjects, the parcellation process resulted in one-piece parcels which were comparable in size (from ~10% to 25% of the total callosal surface), with clear-cut radial boundaries, regularly sampling the whole midsagittal section of the CC along its anterior–posterior axis. The parcellation was not similar but consistent with more classical geometric segmentations (Witelson, 1989), highlighting the large representation of frontal and pericentral projections (Figure 2). It was also successfully applied to the cases of partial agenesis, with the process being robust to the reductional abnormalities, showing which parcels still occupy the residual CC (Figures 3C–H).

### 3.2. Group comparison

#### 3.2.1. FASD vs. controls

**Figure 4:**
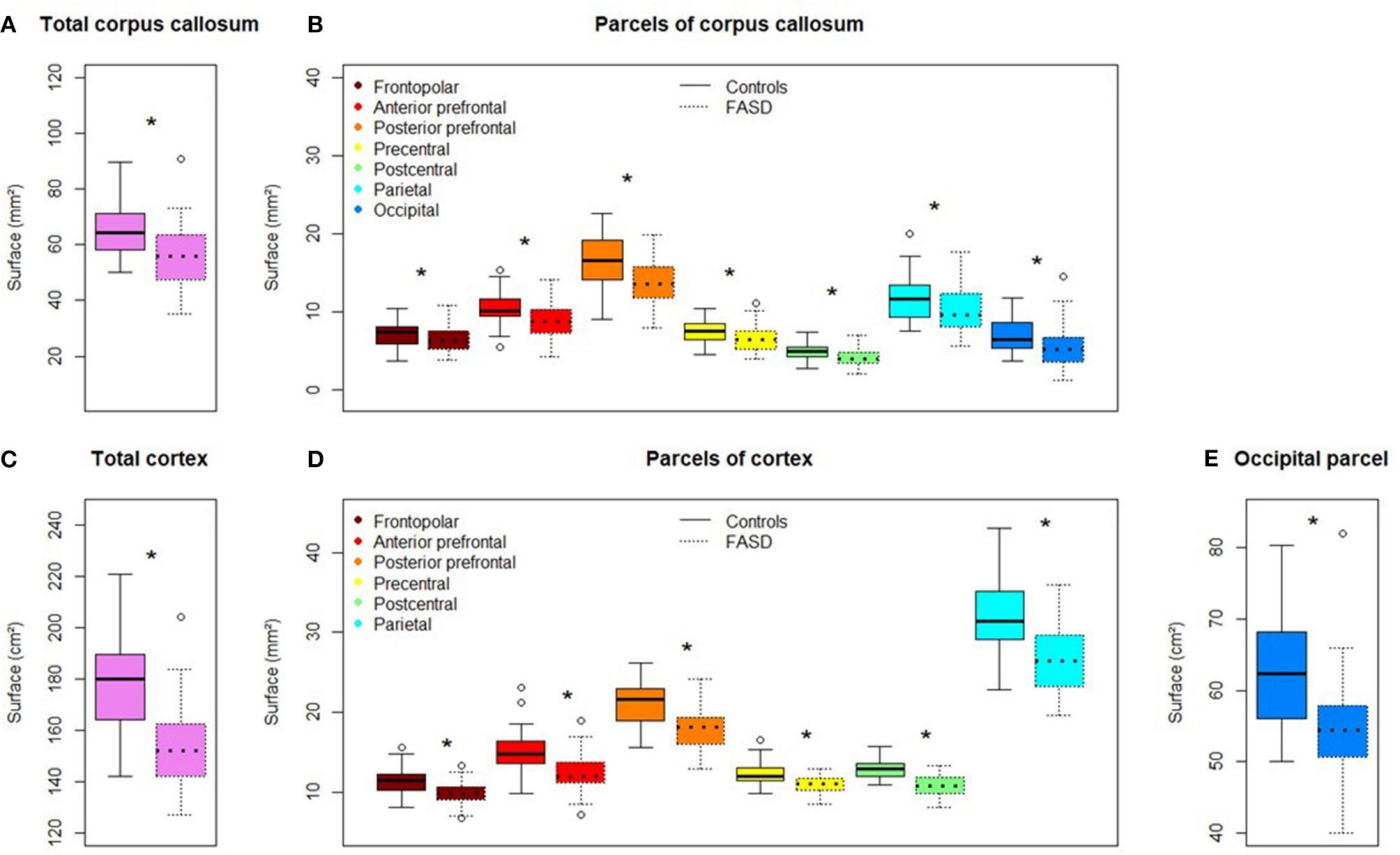
Comparison of callosal and cortical parcel surface areas of controls (left) and subjects with FASD (right, dotted line). **(A)** Total corpus callosum surface area. **(B)** All parcels, right to left: frontopolar, anterior prefrontal, posterior prefrontal, precentral, postcentral, parietal, occipital. **(C)** Total cortical surface area. **(D)** Frontopolar, anterior prefrontal, posterior prefrontal, precentral, postcentral, parietal cortical surface area. **(E)** Occipital cortical surface area. *Significantly different between FASD and control group (FDR corrected *p*-value: *q*-value < 0.05).

Mean callosal and cortical parcel surface areas were all significantly different between FASD and control groups (*q*-value < 0.05) (Figure 4).

**Figure 5:**
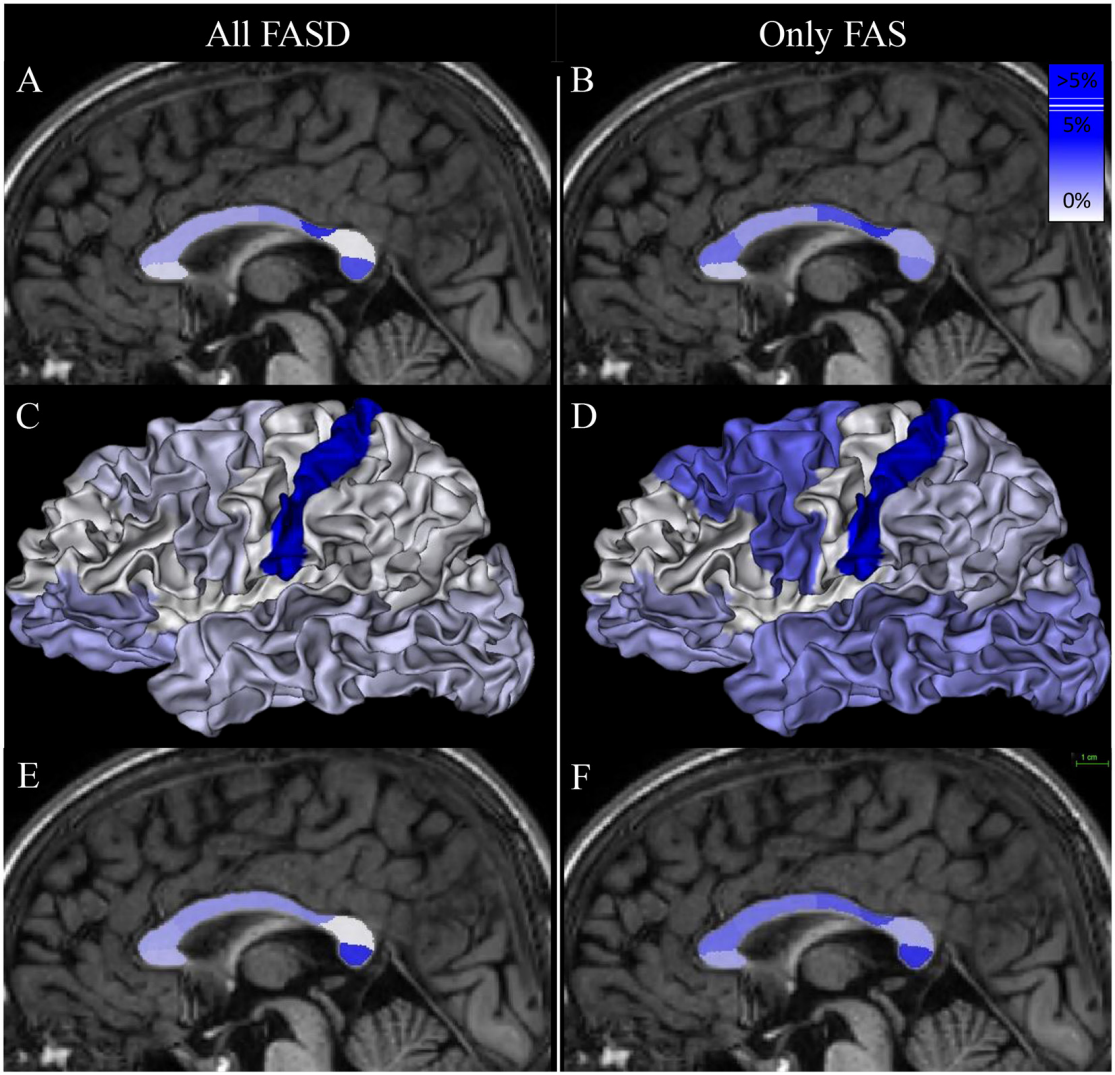
Mapping of the group effect size (FASD vs. control on the left and FAS vs. control on the right) on callosal parcel surface area [**(A, B, E, F)**, respectively] and cortical parcel surface area **(C, D)**. Eta-squared (η^2^) associated with *b*_*DIAG*_ in each model are listed in Tables 3, 4. The scale appears at the top right of **(F)**.

When age, sex, and brain size (TCxS) covariates were included in the analysis of callosal surfaces (*model* 1), a negative effect of FASD was found on the total CC surface, the precentral, postcentral, and occipital CcPS areas (η^2^ = 2.7, 2.3, 6.5, and 4.1%, respectively), that remained significant after FDR correction only for the postcentral one (*q*-value = 0.032). With the same covariates (*model* 2), a strong negative effect of FASD was found on the postcentral %CxPS (η^2^ = 8.9%, *q*-value = 0.007). Adding the corresponding %CxPS to the analysis of each CcPS (*model* 3), a negative effect of FASD was observed on the anterior and posterior prefrontal, the precentral and postcentral CcPS areas, increasing from η^2^ = 1.9 to 2.6%, but not remaining after FDR correction and a stronger negative effect on the occipital CcPS area (η^2^ = 5.7%, *q*-value = 0.014) (Figure 5, Table 3). Note that no effect at all was observed in the frontopolar and parietal callosal or cortical parcels.

**Table 3:** Group effect (FASD vs. control) on callosal parcel surface area (CcPS in models 1 and 3) and cortical parcel surface area (CxPS in model 2).

**All subjects with FASD**	***b***_*****DIAG*****_ **in model 1 (CcPS)**				***b***_*****DIAG*****_ **in model 2 (CxPS)**				***b***_*****DIAG*****_ **in model 3 (CcPS)**			
	η^2^	***t*** **(98)**	***p*** **-value**	***q*** **-value**	η^2^	***t*** **(98)**	***p*** **-value**	***q*** **-value**	η^2^	***t*** **(97)**	***p*** **-value**	***q*** **-value**
Total CC	2.7	−1.90	**0.030**	0.080	-	-	-	-	-	-	-	-
Frontopolar	0.6	−0.81	0.211	0.241	1.4	1.20	0.116	0.333	1.5	−1.36	0.088	0.103
Anterior prefrontal	1.8	−1.49	0.070	0.093	0.0	0.05	0.480	0.492	1.9	−1.73	**0.044**	0.062
Posterior prefrontal	1.8	−1.54	0.064	0.093	0.7	0.89	0.187	0.333	2.2	−1.72	**0.044**	0.062
Precentral	2.3	−1.72	**0.044**	0.088	0.0	0.02	0.492	0.492	2.3	−1.77	**0.040**	0.062
Postcentral	6.5	−2.75	**0.004**	**0.032**	8.9	−3.17	**0.001**	**0.007**	2.6	−1.82	**0.036**	0.062
Parietal	0.0	−0.14	0.445	0.445	0.2	−0.50	0.308	0.431	0.0	−0.04	0.486	0.486
Occipital	4.1	−2.17	**0.016**	0.064	0.8	0.88	0.190	0.333	5.7	−2.87	**0.002**	**0.014**

Eta-squared (η^2^), *t*-value, *p*-value, and q-value (FDR corrected *p*-value) associated with *b*_*DIAG*_ in each model. In bold, *p*-value and FDR-corrected *p*-value (*q*-value) < 0.05. Effect sizes (η^2^) are mapped in Figures 5A, C, E.

#### 3.2.2. FAS vs. controls

In the FAS group, the effect of the FAS diagnosis was also found only on the postcentral CcPS area using (*model* 1) (η^2^ = 12.8%, *q*-value = 0.008). A strong negative effect of FAS was observed on the postcentral CxPS area (η^2^ = 20.4%, *q*-value < 0.001). In *model* 3, the effect of FAS on CcPS followed the same prefrontal to postcentral gradient (from η^2^ = 3.3% to 4.2%) and affected the occipital CcPS (η^2^ = 4.9%, *p* = 0.014, *q*-value = 0.059) but did not remain significant after FDR correction (Figure 5, Table 4).

**Table 4:** Group effect (FAS vs. control) on callosal parcel surface area (CcPS in models 1 and 3) and cortical parcel surface area (CxPS in model 2).

**All subjects with FAS**	***b***_*****DIAG*****_ **in model 1 (CcPS)**				***b***_*****DIAG*****_ **in model 2 (CxPS)**				***b***_*****DIAG*****_ **in model 3 (CcPS)**			
	η^2^	***t*** **(98)**	***p*** **-value**	***q*** **-value**	η^2^	***t*** **(98)**	***p*** **-value**	***q*** **-value**	η^2^	***t*** **(97)**	***p*** **-value**	***q*** **-value**
Total CC	4.6	−2.09	**0.020**	0.069	-	-	-	-	-	-	-	-
Frontopolar	0.9	−0.84	0.202	0.202	1.4	1.03	0.154	0.270	1.8	−1.28	0.103	0.114
Anterior prefrontal	3.2	−1.65	0.052	0.102	0.0	0.05	0.479	0.479	3.3	−1.85	**0.034**	0.059
Posterior prefrontal	2.1	−1.42	0.081	0.108	2.8	1.57	0.061	0.214	3.0	−1.73	**0.044**	0.062
Precentral	4.0	−1.97	**0.026**	0.069	0.1	−0.31	0.378	0.441	3.7	−1.94	**0.028**	0.059
Postcentral	12.8	−3.36	**0.001**	**0.008**	20.4	−4.40	**< 0.001**	**< 0.001**	4.2	−1.98	**0.026**	0.059
Parietal	1.9	−1.30	0.100	0.114	0.8	−0.76	0.224	0.314	1.7	−1.21	0.114	0.114
Occipital	2.8	−1.54	0.064	0.102	1.9	1.18	0.120	0.270	4.9	−2.24	**0.014**	0.059

Eta-squared (η^2^), *t*-value, *p*-value, and q-value (FDR-corrected *p*-value) associated with *b*_*DIAG*_ in each model. In bold, *p*-value and FDR-corrected *p*-value (*q*-value) < 0.05. Effect sizes (η^2^) are mapped in Figures 5B, D, F.

### 3.3. Normative analysis

**Figure 6:**
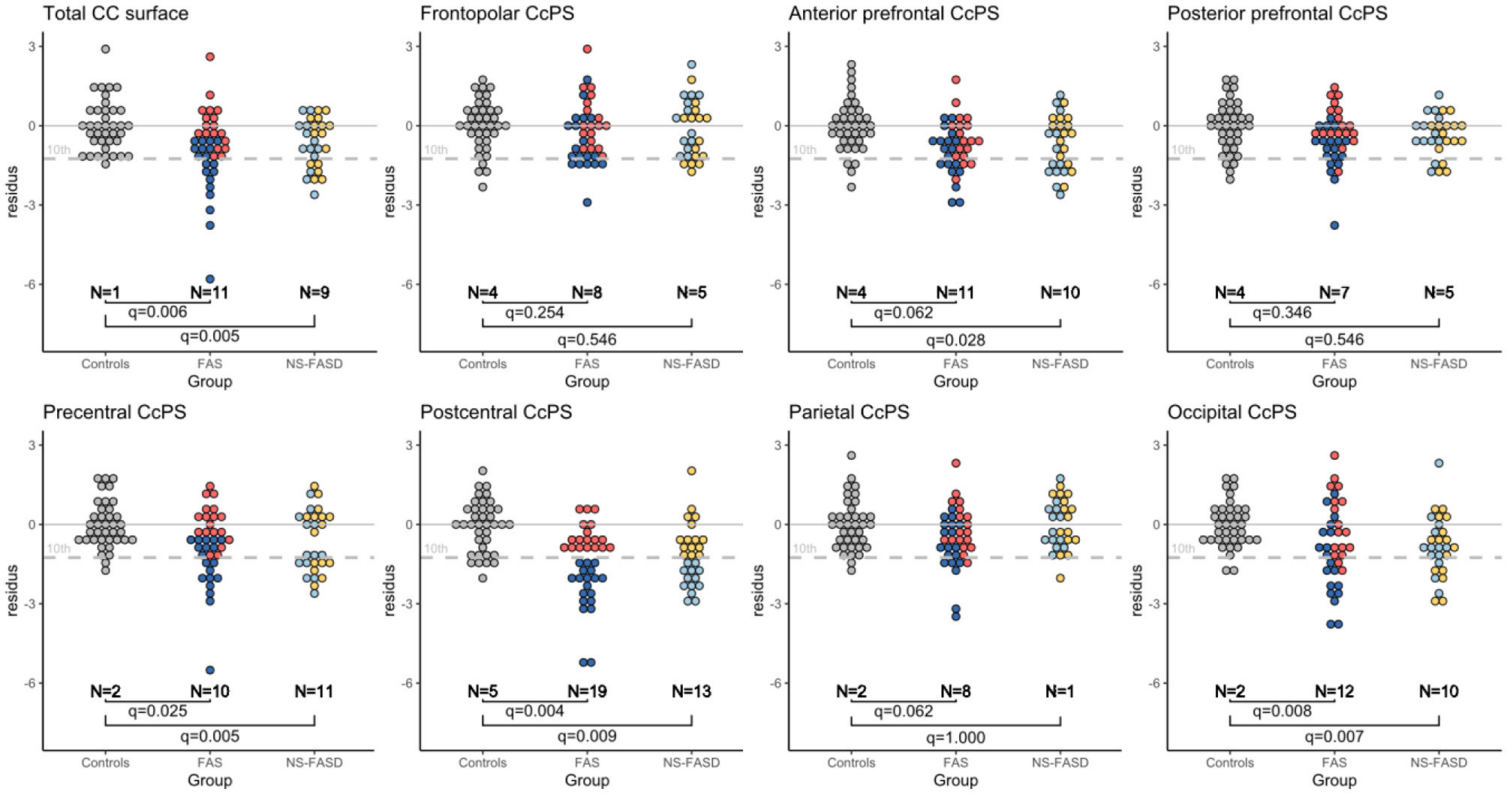
Normative analysis of callosal parcel surface area. Distribution of “residue” of each subject according to regression fitted in the control group, adjusting for age, sex, and brain size (TCxS). Number of subjects below the 10^th^ percentile (dotted gray line) of the control distribution. FDR correction *p*-value of the Fisher test (*q*-value). Subjects identified as “too small” for the postcentral CcPS are tagged in blue so as to be easily identifiable on each plot.

This last step aimed at identifying subjects with one or other of the parcels abnormally small (10^th^ centile threshold) considering the distribution in controls. The distribution of the “residues” from *model 4* fitted in controls (age, sex, and brain size as covariates) is presented in Figure 6, in each group (controls, FAS, and NS-FASD) for each parcel, identifying the subjects below the 10^th^ percentile and comparing their proportion between patients and controls.

Subjects with FAS were significantly in excess below the 10^th^ percentile, for total CC surface (*n* = 11, *q* = 0.006), precentral CcSP (*n* = 10, *q* = 0.025), postcentral CcSP (*n* = 19, *q* = 0.004), and occipital CcSP (*n* = 12, *q* = 0.008) (Figure 6). The two subjects with FAS showing radiologically evident callosal agenesis, whose corresponding CcSP areas were equal to zero, were counted (Figures 3C, D). In a *post hoc* analysis excluding these two subjects, these results persisted except for the precentral CcSP (q>0.05). Subjects with NS-FASD were significantly in excess below the 10^th^ percentile, for total CC surface (n=9, *q* = 0.005), anterior prefrontal CcSP (*n* = 10, *q* = 0.028), precentral CcSP (*n* = 11, *q* = 0.005), postcentral CcSP (n=13, *q* = 0.009), and occipital CcSP (10, *q* = 0.007).

The reduction in the size of the postcentral CcSP was the most frequent. We tagged in blue the subjects presenting with this abnormally small postcentral CcPS on all other parcel plots to see whether they presented or not with other abnormally small CcSP. The subjects with FAS showing an abnormally small precentral CcSP had also too small a postcentral one (dark blue), but it was not the case for all those with an abnormally small occipital CsSP (Figure 6). We can also note that there were more subjects with FASD identified below the 10^th^ for the postcentral CcSP than for the global CC size reduction (20 vs. 32).

## 4. Discussion

Our study compared the morphology of the CC between 65 subjects with FASD and 38 typically developing controls, aged between 6 and 25 years old, by means of a sulci and connectivity-based segmentation of its midsagittal section into seven parcels. We were able to show two main significant spots of surface reduction in the callosal section, one in the peri-isthmic and especially post-isthmic region (precentral and postcentral parcels), the other in the posterior terminal end (occipital parcel). Though mainly posterior, these focal shrinkages were separated by a more preserved section (parietal parcel) and our results suggested progressive damage from the more anterior regions corresponding to the prefrontal parcels to the much reduced post-isthmic region. Furthermore, the hemispherotopic segmentation allowed for an increased understanding of the nature of this callosal section damage showing that the post-isthmic reduction is highly associated with a shrinkage of the corresponding postcentral cortical surface parcel, while, conversely, the very terminal posterior reduction was all the more visible considering the relative preservation of the corresponding occipital–temporal cortical surface parcel. Finally, we suggested how such group results could translate to individual analysis through normative identification of subjects with post-isthmic and terminal callosal parcels that were too small compared to the control distribution, the two markers being not always redundant to each other, and in any case more sensitive than the whole callosal section reduction alone.

### 4.1. Bimodal damage mostly in the posterior half of the corpus callosum

#### 4.1.1. Considering the callosal section only

The surface of the midsagittal section of the CC was globally reduced in our FASD population compared with controls (Figure 4), which is consistent with previous reports, a reduction that affected all the parcels we defined on the anterior–posterior axis. In the first step of our analysis, we searched for an effect that was independent of obvious confounding factors, including age, sex, and a brain size parameter reflecting the amount of hemispheric cortex to be connected through the CC (here TCxS), to detect any heterogeneous damage. The main effect is an anterior–posterior U-shape gradient of surface reduction, bottoming around the isthmus, and is more precisely posterior to the typical narrowing in the postcentral parcel. In fact, this gradient is very probably not unimodal as this first analysis strongly demonstrated another spot of focal surface reduction at the very end of the CC in the terminal occipital parcel, with the parietal parcel being fully preserved in between (Figure 5, Table 3). We found similar results in the FAS-only subgroup even if the terminal reduction was not significant (Figure 5, Table 4).

Hence, we confirmed the previous data of the literature that pointed out a reduction in the midsagittal CC area (Riley et al., 1995; Sowell et al., 2001; Dodge et al., 2009; Jacobson et al., 2017), affecting the posterior isthmic and splenial regions more severely (Sowell et al., 2001; Dodge et al., 2009; Fraize et al., 2022) but demonstrating a more complex landscape of surface reduction in the posterior half of the CC with the preservation of the anterior part of the splenium in between a post isthmic nadir and a terminal splenial dip.

We were able to replicate our previous results (Fraize et al., 2022), which highlighted the narrowing of the isthmus as a recurrent FAS anomaly in a subset of the original MRI dataset, but with a computational automatic imaging approach. Consistently, we showed a major reduction in the callosal surface in the post-isthmic parcel (postcentral) controlling for brain size. At the same time and with simple manual measurements, we failed to show an effect of the FASD on the splenium thickness, incriminating the necessarily noisy measurement of this plump object that is highly variable in shape. The difference with the present study is twofold: First, we have a better proxy of the splenium with surface measurement than only a middle thickness, but more importantly, the splenium is now divided into an anterior part (parietal parcel) and a posterior one (occipital parcel), revealing a differential involvement.

#### 4.1.2. Considering cortical–callosal surface correlations

One main contribution of our callosal parcellation based on sulci landmarks and cortical–callosal connectivity was to allow for the analysis of the focal surface variations of the midsagittal section not only according to the global hemispheric size (TCxS) but more precisely according to the focal extension of the connectivity-related cortical surfaces, with two outcomes for the study.

The primary outcome was that a second step of analysis of the callosal parcel surfaces, including the relative cortical parcel surface as a covariate, emphasized the relative terminal reduction in the callosal section while mitigating the post-isthmic one, both in the FASD group and, to a lesser extent, in the FAS group. Within the limits of statistical power, this analysis also revealed a potential prefrontal-postcentral gradient of relative callosal reduction that could be tackled in a larger study, conducted perhaps at a higher resolution (more parcels or even continuous shape analysis).

A secondary outcome of this parcellation strategy was that, our study, designed to target the CC, revealed a very significant focal cortical surface reduction in FASD and FAS, electively affecting the postcentral gyrus. To our knowledge, this has not been reported yet. The two studies of cortical surface extension in FASD reported discordant observations in other regions: It was decreased in the right temporal surface area in 36 participants with ARND (equivalent to NS-FASD) compared to 52 controls (Rajaprakash et al., 2014), whereas a larger surface area of the right precentral gyrus was observed in a large cohort of exposed subjects but without PAE threshold (Marshall et al., 2022). An indirect insight could also come from gyrification studies: For instance, a reduced sulcal depth was found in the intraparietal sulci, including the postcentral one, of 24 children with FASD correlating with the level of PAE (De Guio et al., 2014), and a reduction in local gyrification was observed in bilateral parietal clusters, including the superior part of the postcentral gyrus and the inferior part of the right one in 30 alcohol-exposed adolescents (Infante et al., 2015). However, in both studies, the postcentral involvement was neither elective nor exclusive, and it would, in any case, only be an indirect correlate of postcentral cortical surface reduction. The heterogeneity of the populations and analyses makes it difficult to put our cortical result further in perspective. Regarding the structure and shape of the CC, the question of the directionality of the observed correlation between the reduction in callosal and cortical parcels also remains open, as it would make sense not only if a defect in interhemispheric connectivity affected the size of the concerned gyrus but also if a primary defect in the cortical extension of this gyrus led to a reduction in the size of the associated callosal sub-bundle.

#### 4.1.3. Insight from preclinical models with callosal damage

The profile of damage in the midsagittal section of the CC that we report should be compared with what has been observed in animal models of PAE, mostly in rodents, even if the choice of species, strain, type of exposure, and measurements are likely to trigger different results (Parnell et al., 2014; Zhang et al., 2019; Milbocker et al., 2022). Interestingly, we found reports not only of global and recurrent terminal reduction in the midsagittal corpus callosum in rats (Moreland et al., 2002) but also of global and middle “isthmic” thinning in mice (O'Leary-Moore et al., 2011), both consistent with our bimodal mostly terminal profile.

### 4.2. Quantitative anomalies of the corpus callosum as a potential diagnostic marker

Pragmatically, we also sought to identify callosal abnormalities that could be found at the individual level. We looked for FASD subjects with callosal parcels that could be considered too small once the appropriate covariates were taken into account. We performed a normative-like analysis by computing the residual for each subject and each callosal surface to the model fitted in the controls while considering all the covariates of interest. We were then able to identify the subjects with FAS or NS-FASD with values below the 10^th^ percentile of the control distribution. Though exploratory due to the limited size of our control group, this individual analysis found global, peri-isthmic (pre and postcentral), and terminal anomalies (< 10^th^ percentile) to be recurrent (in excess) not only in FAS but also in NS-FASD. More interestingly, this individual analysis showed that anomalies in the postcentral or occipital callosal parcel surfaces were more frequent than in the total callosal section alone (for instance in the FAS group, 19+2 vs. 11 subjects), making them better candidate markers of the disease. Besides, if all the patients with FAS showing abnormal precentral values had a postcentral anomaly, this was not the case for the occipital value that tended to add new subjects (Figure 6). This phenomenon appeared to be even more complex in NS-FASD in any case, highlighting the probably multimodal nature of the callosal damage already illustrated by our study.

The question of the specificity of our findings as of any callosal marker in the FASD population is worth raising. Agenesis of the CC is not extremely rare in the general population (0.02%−0.7%) (Glass et al., 2008) and could be as high as approximately 1% in a population of subjects with neurodevelopmental disorders (Jeret et al., 1985). One could therefore expect an additional comparison group of subjects with neurodevelopmental disorders or microcephaly without PAE for a better assessment of specificity. Even in the absence of this type of study, the sheer number of subjects with FAS concerned by at least one peri-isthmic or terminal quantitative anomaly of the CC gives our result a much broader potential for clinical use than visually assessed agenesis of the CC. Indeed, with a reduction in the midsagittal callosal section following the bimodal profile, we found that positively diagnosed FAS could be a relevant feature to strengthen the probabilistic diagnosis of non-syndromic non-specific FASD. In that respect, our result argues for more precise and meaningful neuroanatomical criteria in FASD diagnostic guidelines.

### 4.3. Interest and limitations of the sulci and connectivity-based parcellation

The intrinsic limitations of the purely geometric parcellations in the midsagittal section of the CC, such as Witelson's (Witelson, 1989), have prompted the use of connectivity-based ones, relying on objective and anatomically grounded cortical segmentations (Huang et al., 2005; Styner et al., 2005; Chao et al., 2009; Friedrich et al., 2020). These methods are relevant even when there is no guarantee of the integrity of the extremities, especially the posterior one which is difficult to verify morphologically, and ensure an adequate proxy of inter-individual variations in the relative representation of the different cortical regions at the level of the medial CC. However, there were very few implementations in the field of neurodevelopmental imaging (Lebel et al., 2010), and to our knowledge, our study is the first in a neurodevelopmental condition such as FASD. Relying on the *Hip-Hop* and *MarsAtlas* cortical parcellation, with the homology of the parcels being supported by the reasonable assumption of homology between the large sulci that define the geodesic meridians, our implementation proved to be highly appropriate to sample the anterior–posterior axis of the CC. It also allowed, as previously discussed, the conjoint analysis of cortical and callosal parcels.

Other methodological limitations could be discussed, such as the use of a segmentation tool as *MarsAtlas* initially developed for a healthy adult cohort. The first steps of the *MarsAtlas/Hip-Hop* process rely on the *Morphologist* segmentation pipeline that has currently been successfully used in typically developing or impaired children and adolescents (Borst et al., 2014; Cachia et al., 2016; Kersbergen et al., 2016). Then, the *MarsAtlas* method is essentially based on the identification of homologous sulci in each subject space, without any normalization step. Our cohort was composed of children above 6 years of age whose gyrification has been shown to be very similar to that of adults (White et al., 2010). However, the use of such a method in a pathological population raises the question of the integrity of the main cortical sulci in FASD. Indeed, we lack the knowledge of any reports on FASD, showing that the sulcal pattern may be so impaired as to prevent the use of sulcal landmarks, with the few sulcal modifications reported being mostly quantitative and small-sized (Infante et al., 2015; Hendrickson et al., 2017; Kilpatrick et al., 2021). Besides, the parcellation process was carefully quality checked to avoid mis-segmentation or misclassification of the sulci that would affect the final callosal parcellation (Figure 1). Finally, it should be emphasized that the proposed method is purely object-based morphometry and thus does not rely on any template normalization or averaging in the image domain, which may have unpredictable consequences in pathological populations and raise methodological questions in dysgenetic CC.

Our implementation of this strategy led to a sampling of the midsagittal section of the CC into seven parcels, with subdivisions that proved to be quite relevant in some regions (for instance, isthmus and splenium), but this may still be insufficient. Much more subtle spatial resolution could be achieved with the same rationale using higher resolution cortical atlases, not only in both the large parcels (for instance prefrontal ones) and possibly complex regions, such as the splenium, but also within the thickness of the CC (Park et al., 2008). Provided that a dataset with sufficient statistical power is available, such an increase in spatial resolution and a number of parcels might have revealed a more subtle pattern of surface area reduction.

### 4.4. Other limitations and future directions

With hundred subjects and only a third of typically developing controls, the size of our dataset may have limited the power of the study and the scope of the results, at least because of the risk of sampling bias. However, it can be recalled that the relatively small number of subjects allowed a particularly thorough quality control that would not have been possible with a larger data set, mitigating, in part, the disadvantage of the limitation in group size and that this is a homogeneous monocentric study in terms of both clinical recruitment and MRI acquisitions. However, some potentially interesting results are still exploratory as they did not remain after FDR correction to limit type I risk (for instance, the prefrontal–postcentral gradient) and that, conversely, some interesting small-sized effects may have been missed due to insufficient power.

Our choice of a linear model to regress the data, and in particular, the effect of brain size is debatable since the use of a power model in which the proportions can vary with the size (allometry) (de Jong et al., 2017; Fraize et al., 2023) is more correct and less prone to mismodeling (covering the linear one). The small size of the control group and the low-shared variance share between brain size and callosal section area led us to consider that non-linear modeling was not appropriate for our dataset and to apply only an affine approximation of the scaling effect. A more complex non-linear account for scaling could be used in a replication study with a larger sample.

Last, it is also an understandable but real limitation not to be able to analyze the observed variance with respect to the level of PAE. While PAE is documented to be above a relatively consensual threshold in our population, we do not have further insight into the timing and extent of this exposure.

Hence, in light of our results, we considered that there is a real interest for future studies to implement our segmentation or any similar cortical and connectivity-based one, with a high FAS to NS-FASD ratio to ensure specificity of the findings and a large control group to provide normative data for potential clinical use at the individual level. It could also be of interest to focus on younger samples or even a prenatal population, as to the authors' knowledge, only one ultrasound imaging study has suggested the early damage of the splenium as a diagnostic or prognostic marker of the consequences of PAE in neonates (Bookstein et al., 2005).

## 5. Conclusion

Our study described the surface reduction in the midsagittal section of the corpus callosum in a series of 65 patients with FASD. A unique combination of anatomical and diffusion tensor imaging was used to provide a sulci and connectivity-based parcellation into seven regions, designed to account for the cortical connectivity of the structure and its possible partial agenesis or marked dysgenesis. We demonstrated bimodal damage mostly in the posterior half of the corpus callosum, with a strong post-isthmic narrowing and relative terminal splenium damage, of which only the latter was independent of the reduction in the connected cortical parcel. These anomalies were more frequently and sensitively observed than the reduction in the whole section area and, interestingly, were frequent not only in FAS but also in NS-FASD, opening the field for a clinical application as a diagnostic marker.

## Data availability statement

The datasets presented in this article are not readily available because the data that support the findings of this study are available on request from the corresponding author. The data are not publicly available due to privacy or ethical restrictions. Requests to access the datasets should be directed to JF, justine.fraize@inserm.fr.

## Ethics statement

This study was conducted in accordance with the principles of the Declaration of Helsinki. Subjects' data were studied in accordance with French regulation (MR-004, declaration of conformity n 2059980v0), following approval by the Paris-Saclay research ethics committee (CER-Paris-Saclay-2020-094). Written informed consent from the FASD participants' legal guardian/next of kin was not required to participate in this study in accordance with the national legislation and the institutional requirements. Controls' data were used within the framework of the ethical authorizations of the primary studies (Gene and autism, Inserm C07-33). Written informed consent from the control participants' legal guardian/next of kin was required to participate in the initial study in accordance with the national legislation and the institutional requirements.

## Author contributions

JF, GC, and DG contributed to the conception and design of the study. JF and EK collected and organized the database. GC, FS-M, and YL ensured data processing. JF performed the statistical analysis. JF and DG wrote the manuscript. All authors contributed to manuscript revision, read, and approved the submitted version.
